# The molecular mechanisms of LncRNA-correlated PKM2 in cancer metabolism

**DOI:** 10.1042/BSR20192453

**Published:** 2019-11-12

**Authors:** Ting Tao, Shiyuan Wu, Zheng Sun, Wei Ma, Sichun Zhou, Jun Deng, Qiongli Su, Mei Peng, Gaosheng Xu, Xiaoping Yang

**Affiliations:** 1 Department of Pharmacy, YueYang Maternal-Child Medicine Health Hospital, Yueyang 414000, Hunan, China; 2 Key Laboratory of Study and Discovery of Small Targeted Molecules of Hunan Province, School of Medicine, Hunan Normal University, Changsha 410013, Hunan, China; 3 Key Laboratory of Chemical Biology and Traditional Chinese Medicine Research (Ministry of Education of China), Hunan Normal University, Changsha 410013, Hunan, China; 4 Department of Pharmacy, Zhuzhou Central Hospital, Zhuzhou 412000, Hunan, China; 5 Department of Pharmacy, Xiangya Hospital, Central South University, Changsha 410008, Hunan, China

**Keywords:** cancer targeting, LncRNA, mechanisms, PKM2

## Abstract

Reprogrammed metabolism is an important hallmark of cancer cells. Pyruvate kinase (PK) is one of the major rate-limiting enzymes in glucose metabolism. The M2 isoform of PK (PKM2), is considered to be an important marker of metabolic reprogramming and one of the key enzymes. Recently, through the continuous development of genome-wide analysis and functional studies, accumulating evidence has demonstrated that long non-coding RNAs (LncRNAs) play vital regulatory roles in cancer progression by acting as either potential oncogenes or tumor suppressors. Furthermore, several studies have shown that up-regulation of PKM2 in cancer tissues is associated with LncRNAs expression and patient survival. Thus, scientists have begun to unveil the mechanism of LncRNA-associated PKM2 in cancer metabolic progression. Based on these novel findings, in this mini-review, we summarize the detailed molecular mechanisms of LncRNA related to PKM2 in cancer metabolism. We expect that this work will promote a better understanding of the molecular mechanisms of PKM2, and provide a profound potential for targeting PKM2 to treat tumors.

## Introduction

In the field of tumor research, much attention has recently been paid to the metabolic reprogramming of tumor cells. A key feature in metabolic reprogramming is glucose metabolism—an important life process that provides energy and nutritional resources for organisms and their individual cells. A characteristic that differentiates cancer cells from normal cells is metabolic regulation, with a hallmark of tumor cells being a shift from oxidative phosphorylation to glycolysis-dependent metabolism, even in aerobic conditions. Cancer cell metabolism is characterized by increased glycolysis and lactic acid production instead of increased aerobic respiration. This phenomenon is known as aerobic glycolysis or the Warburg effect [1]. Pyruvate kinase (PK) is one of the main rate-limiting enzymes and physiologically irreversible step in glycolysis, which converts phosphoenolpyruvate (PEP) into ADP by the phosphate group transfer to produce adenosine triphosphate (ATP) and pyruvate [2]. PK has four isoforms: L, R, M1 and M2. PKL is mainly expressed in the liver, while PKR in red blood cells; both are encoded by PKRl. PKM1 is expressed in many types of normal tissue, whereas M2 isoform of PK (PKM2) is particularly expressed in highly proliferating cells; both isoforms are encoded by PKM [3]. PKM2 is an important rate-limiting enzyme in glycolysis during glucose metabolism.

## The function and regulation of PKM2

In the early 1980s, the functional study of PKM2 was initiated by the identification of a candidate gene in mouse [4]. Noguchi et al. [5] showed that PKM1 and PKM2 are encoded by the same PKM gene. The alternative splicing of *PKM* mRNA produces PKM1 or PKM2, which depends on the mutually exclusive selection of exon 9 or 10 [6]. A switch from the PKM1 to PKM2 of mRNA splicing is enhanced by c-Myc oncogene, it indicated that cancer cells actively engage in this transformation to meet their needs for proliferation and metabolism [7]. In addition, the change of the PKM1 to the PKM2 isoform in various cancers is based on the study of the relative abundance of PKM1 and PKM2 in normal and tumor tissues [8,9]. PKM2 isoform oscillates from high to low PK activity (that is, from tetramer to dimer form). The dimeric form of PKM2, also known as low PK activity, can produce several glycolytic intermediates that serve as building blocks for the biosynthesis of amino acids, lipids and nucleotides. During the expression of spliced PKM1, that the PKM2 deletion is inhibited by controlling exon 9 usually maintains low PK activity induced by normal growth signal [10]. Zhou et al. [11] found that PKM2 levels can serve as a predictor to determine whether a cancer is non-invasive or highly aggressive. Based on this, the dominance of dimeric form of PKM2 provides a metabolic advantage for cancer cell proliferation.

PKM2, when activated by PEP and PEP or fructose 2,6-bisphosphate, converts PEP into ADP by transferring phosphate group, thereby producing ATP and pyruvate to catalyze the physiological irreversible step in glycolysis [12]. During this process, PEP and ATP bind to the active site with a mechanism promoted by cationic K^+^ and Mg^2+^ or Mn^2+^ [13,14]. The PKM gene encodes the PKM1 and PKM2 isoforms. Although PKM1 and PKM2 catalyze the same reaction in glycolysis, they also differ in that PKM1 has constitutively high catalytic activity, while PKM2 is allosterically regulated [15]. By the virtue of constinuous study of the allosteric regulation of PKM2, accumulating evidence suggests that ATP, oxalate, alanine and phenylalanine can act as allosteric inhibitors of PKM2. These inhibitors block the allosteric regulation of PKM2 through various mechanisms [16–18]. In contrast, serine, a specific PKM2 activator, often binds to each monomer to promote the formation of the active tetramer [19]. Furthermore, it has been reported that SAICAR (succinylaminoimidazolecarboxamide ribose-5′-phosphate), intermediate product through the process of *de novo* purine nucleotide synthesis, which specifically stimulates PKM2 and then promotes cancer cell survival [19].

### PKM2 post-translational modifications

#### Phosphorylation

Phosphorylation, one of the major post-translational modification of PKM2, either activates or inactivates PKM2. It was found that PKM2 is phosphorylated by Tyr^105^ via oncogenic tyrosine kinase or combined with tyrosine phosphorylation peptide after growth factor intervention, which reduces the pyruvate activity of PKM2 and change energy production to anabolic processes during glucose metabolism [20]. Cheng et al. [21] found that PKM2 directly phosphorylated PAK2 at Ser^20^, Ser^141^ and Ser^192/197^ via binding and kinase assays *in vitro*. Further research has shown that knocking out PAK2 in PDKM2 overexpressing PDAC cells diminished mobility *in vitro* in cell and metastatic ability *in vivo*. The phosphorylation of PKM2 on Thr^454^ increases A549 proliferation and decreases mitochondrial function [22]. In addition, Chen et al. [23] proved that tripartite motif-containing protein 35 (TRIM35) through the blockade of PKM2 Y105 phosphorylation suppresses the tumor progression of hepatocellular carcinoma (HCC) cells.

#### O-GlcNAcylation

O-GlcNAcylation is a post-translational modification involving the attachment of O-linked N-acetylglucosamine (O-GlcNAc) moieties to cytoplasmic, nuclear and mitochondrial proteins to regulate fundamental cellular processes in metazoans [24]. The science in 2012 years has reported that phosphofructokinase 1 (PFK1), as a major regulatory enzyme controls flux through glycolysis, which has been shown to regulate cell metabolism via O-GlcNAcylation [25]. Recently, Singh et al. [26] found that O-GlcNAcylation promotes aerobic glycolysis and tumor growth by inhibiting PKM2 catalytic activity. Wang et al. [27] identified that a post-translational modification on PKM2, O-GlcNAcylation, which destabilized the active tetrameric PKM2, reduced PK activity, and led to nuclear translocation of PKM2.

### Other post-translational modifications

In addition to phosphorylation and O’GlcNAcylation, which regulates the activity of PKM2, PKM2 can also be modified by other post-translational modifications, including acetylation [28], succinylation [29] and methylation [30]. For instance, under normal glucose conditions, deacetylated hnRNP A1 reduced PKM2 in HCC cells and increased PKM1 alternative splicing, resulting in decreased metabolic activity of PK [31]. At the same time, succinylation of PKM2 at Lysine 498 (K498) increases its activity [32].

### The regulation of PKM2 by signaling pathway

Several signaling pathway and transcription factor have been reported to regulate the expression of PKM2. PKM2 induces downstream metabolism and proliferation-related gene expression by activating transcriptional cofactors of hypoxia-inducible factor 1 α (HIF-1α) and β-catenin [33]. It acts as a protein kinase in the nucleus and directly phosphorylates STAT3 and histone H3 to promote tumor transformation [34,35]. The phosphoinositide 3-kinase (PI3K)/mammalian target of rapamycin (mTOR) signaling pathway is found to be activated in the tumor cells. mTOR, as a central activator of the Warburg effect, which induces PKM2 in many kinds of human tissues and cell lines. Furthermore, inhibition of mTOR also reduces the expression of PKM2 [36]. Our previous studies showed abnormally high expression of PKM2 in bladder cancer and elucidated the molecular mechanism of its regulation of the rapid proliferation and drug resistance of the cancer [37,38]. Based on these findings, PKM2 has been identified as a potential target for cancer diagnosis and treatment.

## The role of PKM2 in cancer metabolism

A significant feature of cancer cell metabolism is the increase in glycolysis. PKM2, as an important rate-limiting enzyme in glycolysis, plays an important role in tumor metabolism. In the last decade, more and more researches have focused on the metabolism of PKM2 in tumor. On one hand, PKM2 increases the synthesis of lactate and macromolecules to promote tumor proliferation via exhibiting low PK activity and nuclear moon-lightening functions. On the other hand, PKM2, not only can act as a protein kinase but as a transcriptional co-activator of genes, involved in cell proliferation, migration and apoptosis. PKM2 leads to increases in glucose utilization [39] and changes in redox balance [40] of tumor cells when it is up-regulated. Thus, PKM2 serves multiple functions that are closely related to cancer development and metastasis. Based on these findings, PKM2 is positioned as a potential target for cancer therapy.

## Long non-coding RNAs

Long non-coding RNAs (LncRNAs) are defined as transcripts longer than 200 nucleotides that are not translated into protein [41]. They can monitor immune cell development [42] and other biological processes, possibly by interacting with proteins and RNAs [43]. In recent years, there have been accumulating reports of dysregulated LncRNA expression in various cancers, suggesting that LncRNAs may act as carcinogenic or anti-cancer factors [44]. Furthermore, it has been shown that LncRNAs are key regulators of cancer metabolism [45,46], and several LncRNAs have been implicated in the development of cancer, including LINC01554, LINC00689, MAFG-AS1, highly up-regulated in liver cancer gene (HULC), FEZF1 antisense RNA 1 (FEZF1-AS1), lincRNA-p21, MEG3, HOXB cluster antisense RNA 3 (HOXB-AS3), tumor protein 53 target gene 1 (TP53TG1) and lncRNA regulator of reprogramming (linc-RoR) [47]. Interestingly, the LncRNA CRNDE has been shown to promote metabolic changes by switching the metabolism of cancer cells to aerobic glycolysis [48], establishing a novel relationship between LncRNA and glycolysis.

However, the detailed molecular mechanisms involved in LncRNAs and glycolysis remain unclear. In this mini-review, we summarize several novel molecular mechanisms in which LncRNAs are associated with PKM2 in cancer metabolism. We expect that a better understanding of the various underlying molecular mechanisms will help clarify the role of PKM2 in tumors, establishing a solid mechanistic foundation for tumor therapy targeted at PKM2.

## LncRNAs associated with PKM2 expression

### HULC

The gene that is most up-regulated in HCC is *HULC*, a novel mRNA-like LncRNA. Emerging evidence suggests that HULC accelerates the development of hepatocarcinogenesis and deregulates lipid metabolism in HCC cells [49]. It is expected that HULC has the potential to become a cancer biomarker.

It is well established that metabolic reprogramming of cancer cells is driven by the activation of oncogenes or the loss of tumor suppression. Phosphate and tension homology deleted on chromsome ten (PTEN), a tumor suppressor gene, was identified by three groups in 1997 [50]. Chen et al. found that PTEN overexpression inhibited the phosphorylation of protein kinase B (AKT) and reduced the expression level of PKM2 [51]. PTEN was also shown to play a critical role in regulating the proliferation of cervical cancer cells [51]. In a recent study, Xin et al. [52] reported that HULC activates the PI3K/AKT/mTOR pathway in human liver cancer by inhibiting the expression of PTEN, depending on autophagy. They discovered that abnormally high expression of HULC in liver cancer cells reduced the expression of PTEN and increased the expression of PKM2 [53].

There is accumulating evidence that LncRNAs in competitive endogenous RNA (ceRNA) networks play a key role in tumor survival. MicroRNAs silence genes through binding to mRNAs and ceRNAs [37]. Wang et al. [54] reported evidence that HULC acts as an endogenous ‘sponge,’ inhibiting the activity of miR-372 through a ceRNA that induces the translational de-repression of PRKACB. Furthermore, miR-372 enhances the expression and activity of PKM2 via the β-catenin–LEF/TCF4 pathway, implying it has a critical role in bridging the relationship between PKM2 and HULC [55]. These results have revealed a new mechanism for interactions involving RNAs and the HULC/PKM2 axis. This cell signaling pathway could provide a novel focus for exploring therapeutic candidates for cancer treatment.

### LincRNA-p21

Long intergenic non-coding RNA (LincRNA)-p21, one of the LncRNAs regulated by p53, is a repressor of p53-dependent transcriptional responses [56]. There is increasing evidence that lincRNA-p21 plays vital roles in various cancers. In microglia cells, the overexpression of lincRNA-p21 was found to be sufficient to increase PKC-δ, which in turn further enhanced the expression of p53 and lincRNA-p21, resulting in lipopolysaccharide-induced p53/lincRNA-p21 expression [57]. LincRNA-p21 regulates the proliferation and apoptosis of vascular smooth muscle cells by activating the transforming growth factor β 1 (TGF-β1) signaling pathway, and it plays a role in thoracic aortic aneurysms [58]. Interestingly, when PKM2 was knocked down, this substantially inhibited the tumorigenesis and proliferation of prostate cancer cells in which lincRNA-p21 had been silenced [59].

There is clear evidence of associations between PKM1 and PKM2 and the PI3K/AKT/mTOR pathway [60]. For example, it has been reported that the PI3K/AKT/mTOR cascade is a stimulatory factor for PKM2 and an important oncogenic signaling pathway in prostate cancer [61]. Sun et al. [62] discovered that, under normal conditions, mTOR was a major positive regulator of the Warburg effect in both cancer and benign tumor cells. *In vitro* and *in vivo* studies have confirmed that mTOR can act as a positive regulator of PKM2 expression. For example, kidney tumor samples from a tuberous sclerosis complex mouse model (using Tsc2^del3/+^ mice), in which the expression of mTOR had increased, exhibited robustly elevated PKM2 expression [62]. Consistent with this, PKM2 expression was shown to be reduced when human malignant cell lines were treated with rapamycin, a potent inhibitor of mTOR [63]. Our group recently discovered that the phosphorylation of both AKT and mTOR was significantly reduced when PKM2 was knocked down or following treatment with shikonin, a PKM2 inhibitor [38]. When the expression of PKM2 was normal or silent, the levels of phosphorylated AKT and mTOR decreased under the action of the AKT inhibitor MK2206, revealing a novel relationship between PKM2 and the AKT pathway [38].

Yang et al. [64] demonstrated that the induction of lincRNA-p21 by hypoxia is an essential part of hypoxia-enhanced glycolysis. They found that low levels of lincRNA-p21 significantly reversed the cellular metabolic changes induced by hypoxia. To explore the relationship between glycolysis and lincRNA-p21, Wang et al. [65] established a xenograft tumor model in nude mice by injecting them with lincRNA-p21-silenced DU145 and LNCaP prostate cancer cells that expressed PKM2 (or control) shRNA. They found that the inhibition of PKM2 significantly reduced tumorigenesis and the progression of the LNCaP cells. Consistent with this, the tumorigenic capacity of the lincRNA-p21 DU145 cells was blunted after targeting PKM2 [65]. In addition, the overexpression of lincRNA-p21 in DU145 and LNCaP cells reduced the level of PKM2, confirming that lincRNA-p21 is a negative regulator of PKM2. These results demonstrate that the regulatory mechanism of PKM2 mediated by LincRNA-p21 depends on the PTEN/AKT/mTOR cascade [65].

Gain-of-function p53 mutations often occur in human tumors, promoting tumor development. In a comprehensive analysis of nucleotide levels, Stone et al. [66] showed that PKM2 knockdown reduced the relative levels of N-carbamoyl aspartate, UTP and dTTP, and impaired the incorporation of glucose-derived carbon into UTP, whereas silencing p53 increased the levels of these metabolites. According to the results of a genome-wide CRISPR/Cas9-based loss-of-function proliferation rescue screen in PKM2-silenced cells, Kim et al. [67] found that the *p53* gene, a tumor suppressor, scored the top one hits among more than 20000 genes detected. Furthermore, they further demonstrated that PKM2 inhibited the expression and function of p53 in proliferating endothelial cells (ECs), preventing it from inducing growth arrest at multiple points in the cell cycle [67]. Of interest, lincRNA-p21 has been shown to inhibit many genes in a p53-dependent transcriptional response [68]. Targeting glycolysis could be a therapeutic strategy for prostate cancer patients with low expression of lincRNA-p21. Wang et al. [67] also found that PKM2 expression, glucose consumption and lactate production remained unchanged when p53 was overexpressed in DU145 cells in which lincRNA-p21 was silenced by shRNA, which indicated that lincRNA-p21 negatively regulated aerobic glycolysis independent of p53 signaling.

### LncRNA maternally expressed gene 3

LncRNA maternally expressed gene 3 (MEG3) is a maternal imprinting gene which is located at human chromosome 14q32. Its length is 85, approximately 1.6 kb [69]. Surprisingly, in contrast with other LncRNAs, there is increasing evidence that LncRNA MEG3 is down-regulated in various types of tumors, and low levels of MEG3 are associated with poor prognosis in cancer patients [70]. MEG3 may therefore play an important role in the development of cancers.

The overexpression of MEG3 has been shown to promote the osteogenic differentiation of mesenchymal stem cells in patients with multiple myelomas through targeting BMP4 transcription [71]. Wu et al. [72] reported that double mutant p53 (N340Q/L344R) promoted hepatocarcinogenesis mediated by PKM2. MEG3 regulates genes in the TGF-β pathway by forming RNA–DNA triple structures [73]. TGF-β is a cytokine that mediates a variety of cellular responses, including the suppression of cell proliferation and reaction of differentiation, ageing and apoptosis [74]. Chen et al. [75] found, after PKM2 knockdown in HCC cells, not only increases in the expression of the TGF-β receptor 1 gene and Smad2/3 phosphorylation levels, but also a significant decrease in the phosphorylation of Smad2 in HCC. These results suggest close associations between PKM2 and the Smad and TGF-β pathways during HCC migration and invasion processes.

PKM2 has a critical function in tumor metabolism and growth [76]. In addition, it has been reported that PKM2 plays crucial roles in the growth, survival and migration of tumors [77]. Importantly, miR-122 regulates tumor metabolism, reversing drug-fastness and hepatotoxicity damage in HCC [78]. Zheng et al. [79] for the first time demonstrated that, in hepatocarcinogenesis, MEG3 negatively regulated the activity of PKM2 and the β-catenin signaling pathway as a tumor suppressor, reducing the expression and nuclear location of PKM2 in an miR-122-dependent fashion. Furthermore, they showed that the overexpression of MEG3 enhanced the expression of CTCF, CREB, H3K9me3, H3K36me3, and RNA polII in the miR-122 promoter region, as well as significantly increasing the loading of pre-miR-122 and mature miR-122. Importantly, miR-122 inhibited the activity of luciferase bearing the PKM2 3′-untranslated region (UTR), demonstrating that miR-122 can target the 3′-UTR of PKM2 and reduce its expression [79].

### Linc-RoR

Linc-RoR was originally identified as a reprogrammable regulator of induced pluripotent stem cells [80], but in recent years there have been numerous reports of the molecular mechanisms of linc-RoR in tumors. Lnc-RoR regulates the expression of core transcription factors by acting as a microRNA sponge in the self-renewal of human embryonic stem cells [81]. Furthermore, linc-RoR can promote estrogen-independent growth of estrogen receptor (ER)-positive breast cancer by activating ERK, which in turn phosphorylates ERs at Ser^118^, promoting the ligand-independent activation of ERs [82]. Lnc-RoR is dramatically unregulated in triple-negative breast cancer, promoting cell invasion via the miR-145/ARF6 pathway [83]. Interestingly, Li et al. [84] reported that inhibiting linc-RoR in pancreatic cancer could reduce the expression of PKM2, partly through switching PKM2 to PKM1. This switch was evidenced by targeting polypyrimidine tract bindings (PTBs), which are pivotal regulators of PKM splicing. Interestingly, a fluorescence *in situ* hybridization assay demonstrated that linc-RoR and miR-124 interacted directly. Furthermore, miR-124 overexpression significantly reduced levels of PTBP1, suggesting that PTBP1 might be critical in the potential relationship between miR-124 and PKM2 [84].

Lnc-RoR can also regulate the expression of target genes using the ceRNA mechanism. Takahashi et al. [85] reported that linc-RoR cannot directly interact with HIF-1α mRNA because it has no sequence complementary to HIF-1α. However, linc-RoR can be used as a ceRNA to adsorb miR-145; it then mediates the expression of HIF-1α mRNA through miR-145 [86]. Based on the results of chromatin immunoprecipitation assays, Luo et al. [87] confirmed that PKM2 bound directly to HIF-1α and not to HIF-2α, demonstrating the support favoring an interaction between PKM2 and HIF-1α. Li et al. [88] further reported that reactive oxygen species (ROS) promoted insulin-induced glycolysis and PKM2 expression in HCC, indicating that insulin up-regulates PKM2 expression in an ROS-dependent manner via miR-145 suppression. Interestingly, based on the results from 31 pancreatic ductal adenocarcinoma tissue samples paired with adjacent normal tissue, Chen et al. [84] reported a negative correlation between linc-RoR and miR-124 levels, and that the presence of two 8-mer binding sites among miR-124 and linc-RoR may be critical for this. Thus, the linc-RoR/miR-124/PKM2 axis may provide a novel molecular pathway for exploring the relationship between lincRNA and PKM2 in tumors.

### LncRNA FEZF1-AS1

FEZF1-AS1 is an LncRNA recently identified via LncRNA microarrays in colorectal cancer (CRC). Analysis of the FEZF1 gene sequence has shown that it is located on chromosome 7 of the FEZF1 opposite strand [89]. FEZF1-AS1 has been shown to play a key role in the proliferation and growth of gastric cancer cells via transcriptional activation of the K-ras gene [90].

Wang et al. reported that down-regulation of FEZF1-AS1 repressed the migration and invasion of HCC cells through the inhibition of JAK2/STAT3 axis-mediated epithelial–mesenchymal transition [91]. Interestingly, our group has demonstrated that silencing PKM2 inhibited STAT3 phosphorylation, suggesting a close association between PKM2 and STAT3 [37]. Recently, Ye et al. [92] reported that reducing the expression of FEZF1-AS1 or ZNF312b inhibited glycolysis, glucose uptake and lactic acid production in prostate cancer cells, indicating a relationship between the FEZF1-AS1/miR-107/ZNF312B pathway and the Warburg effect. FEZF1-AS1 can bind PKM2 protein, increasing its stability. Increased cytoplasmic and nuclear PKM2 levels have been shown to promote PK activity and lactate production, with FEZF1-AS1-induced nuclear PKM2 up-regulation in turn activating STAT3 signaling [93]. Thus, FEZF1-AS1/PKM2/STAT3 signaling could be a promising novel therapeutic target in cancer treatment.

### Other LncRNAs

MAFG-AS1, a novel LncRNA, is located in the region of chromosome 17q25.3 in which some differentially expressed genes of are embedded [94]. It shares a head-to-head promoter with MAFG, which induces the methylator phenotype of CpG islands and CRC tumorigenesis [95]. Recently, Cui et al. [96] reported LncRNA MAFG-AS1 to be a novel oncogenic LncRNA that inhibited the growth and development of CRC. The underlying mechanism for this effect may be that it acts as a sponge for miR-147b, resulting in the down-regulation of PDK1, PFK1 and PKM2 [96].

TP53TG1, a p53-induced LncRNA, has been shown to play a key role in the development of human cancer [97]. The knockdown of TP53TG1 significantly inhibited the migration ability of U87 cells under treatment with various levels of glucose, whereas the overexpression of TP53TG1 enhanced the expression of IDH1 and reduced the expression of PKM2 in glioma cells [97]. These results suggest that TP53TG1 may promote cell proliferation and migration by affecting the expression of genes related to glucose metabolism in glioma cells when there is glucose deficiency [98].

Recently, Huang et al. [99] reported that HOXB-AS3 encodes a conserved 53-amino acid small peptide, previously considered to be a novel human LncRNA. They found a positive correlation between levels of the HOXB-AS3 peptide and levels of PKM2 mRNA in sample tissues, but a negative correlation with PKM1 [99]. The HOXB-AS3 peptide suppressed CRC growth through blocking hnRNPA1-mediated PKM splicing, thereby inhibiting the formation PKM2 and suppressing the reprogramming of the glucose metabolism; this suggests that PKM2-associated HOXB-AS3 has a role in tumor formation and progression [99].

LINC01554 was first defined as a tumor suppressor in HCC, whose downexpression could serve as potential prognostic biomarker for HCC patients [100]. Zheng et al. [100], by *in vitro* and *in vivo* functional assays, indicated that LINC01554 exerted tumor-suppressive function and blocked aerobic glycolysis. Furthermore, they further showed that LINC01554 down-regulation empowers cancer cells acquiring high aerobic glycolysis to sustain cells growth advantages via regulating PKM2 and Akt/mTOR signaling pathway [100]. In previous studies, it reported that LINC01554 was associated with fibrosis in patients with nonalcoholic fatty liver disease (NAFLD) and survival of esophageal carcinoma patients [101,102].

As another representative, LINC00689 was recognized as an obesity-related gene in Northern Han Chinese in previous study [103]. The recent study revealed that LINC00689 is a LncRNA identified to be markedly elevated in glioma to normal brain tissues. In U251 and U87 cells, both *in vitro* and *in vivo* assays demonstrated that LINC00689 promotes the proliferation, migration, invasion and glycolysis via directly interacting with miR-338-3p to promote PKM2 expression. In addition, Liu et al. [103] demonstrated that the knockdown of LINC00689 represses the proliferation, migration, invasion and glycolysis of both U251 and U87 cells, while PKM2 expression was restored in U251 cells knocked down by LINC00689.

## Discussion

**Figure 1:**
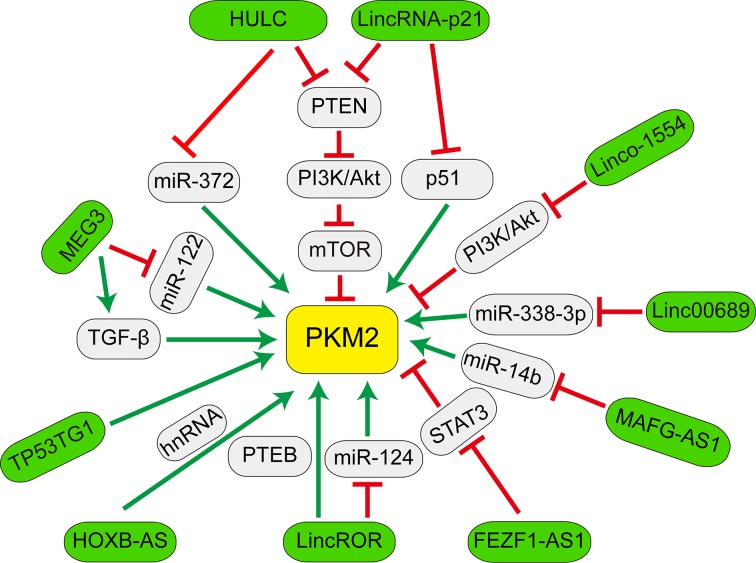
Schematic illustration of LncRNA-correlated PKM2 regulation network On the one hand, several LncRNAs (green) regulate PKM2 expression via affecting cell signaling pathways (blue). (1) HULC and LincRNA-p21 activate the PI3K/AKT/mTOR pathway in human liver cancer by inhibiting the expression of PTEN and PKM2. Furthermore, LINC01554 down-regulation empowers cancer cells acquiring high aerobic glycolysis via regulating PKM2 and Akt/mTOR signaling pathway. (2) LincRNA-p21 negatively regulated aerobic glycolysis independent of p53 signaling. (3) MEG3 regulates genes in the TGF-β pathway by forming RNA–DNA triple structures. In additional, PKM2 knockdown increases the expression of the TGF-β1 gene in HCC cells. MEG3 promotes the expression and maturation of miR122 which targets PKM2. On the other hand, several LncRNAs such as HULC, MEG3, lincROR, MAFG-ASI and LINC00689 regulate PKM2 expression by targeting miRNAs (gray).

Metabolic reprogramming has been widely recognized as a functional marker of cancer cells and plays an extremely important role in growth and proliferation of cancer cells. Therefore, exploring metabolic reprogramming may be an efficient approach to explore potential targets in cancer treatment. PKM2 has been shown to have a critical role in cancer cell metabolism. Revealing functions and regulatory effects of PKM2 have markedly improved our understanding of central role of PKM2 in tumor growth in the past decade. Furthermore, compelling evidence regarding the expression, localization, post-translational modification and allosteric regulation of PKM2 has been collected. With the rapid development of high-throughput analysis and gene editing, evidence from whole genome and transcriptome sequencing suggest that the complexity of an organism may be precisely regulated by short or LncRNAs which belong to the noncoding portions of the genome [104]. In this mini-review, we summarized the recent understanding of the molecular mechanisms of PKM2-associated LncRNAs, including MAFG-AS1, HULC, FEZF1-AS1, LincRNA-p21, MEG3, HOXB-AS3, TP53TG1 and Linc-ROR in cancer metabolism. Simultaneously, we also deliberate how LncRNAs regulate the functions of PKM2 and which pathways are involved in these processes, shown in Figure 1.

As discussed above, major molecular mechanisms are involved in the regulation of PKM2 by LncRNA in cancer development and treatment, which are classified into conventional molecular signaling pathways including PTEN-PI3K-Akt-mTOR and P53, protein alternative splicing, and miRNA-associated ceRNA mechanisms. PTEN-PI3K-Akt-mTOR is one of the most typical cell metabolism-associated pathways.

**Figure 2:**
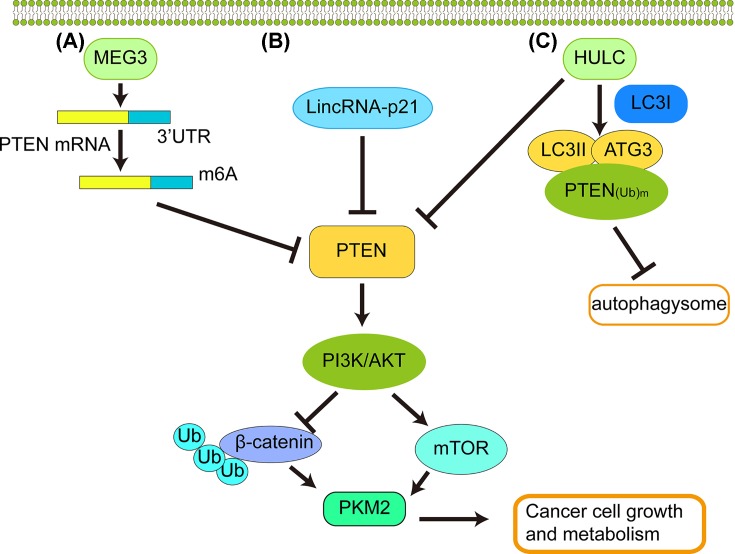
LncRNAs regulate PKM2 expression to inhibit cancer cell metabolism via PTEN and PI3K/AKT/mTOR signal pathway (**A**) MEG3 promotes β-catenin degradation through a PTEN-dependent ubiquitin–proteasome system. Thus, MEG3 inhibits β-catenin activity through PKM2 reduction and PTEN increase. (**B**) LincRNA-p21 is a negative regulator of PKM2. The regulatory mechanism of PKM2 mediated by LincRNA-p21 depends on the PTEN/AKT/mTOR cascade. (**C**) When HULC is overexpressed, HULC increases autophagy by increasing the expression of LC3I and LC3II. At the same time, HULC enhances the interaction between LC3 and ATG3. Thus, HULC inhibits PTEN through enhancing the cellular autophagy by increasing ubiquitin–proteasome dependent LC3II. The result is activation of the AKT-PI3K-mTOR pathway to inhibit tumor cell growth and metabolism when PTEN is reduced, based on inhibition of PTEN in liver cancer cells. Strikingly, HULC reduced the expression of PTEN, β-catenin and increased the expression of PKM2 in hematoma cells.

As mentioned above, HULC, one of important LncRNAs, reduces the expression of PTEN and enhances the expression of PKM2 in liver cancer cells. The activated PI3K-AKT-mTOR pathway by HIF-1-induced transcriptional enhancement and c-Myc-dependent hnRNPs increases the expression of PKM2 [62]. PKM2 is an important target molecule of mTOR downstream, and excessive activation of mTOR will promote the expression of PKM2. The studies have reported that down-regulation of PKM2 expression can attenuate cell tumorigenicity caused by mTOR activation [105]. As shown in this review, LincRNA-p21 is a negative regulator of PKM2 and this regulatory mechanism is dependent on PTEN/AKT/mTOR cascade. In contrast, HULC activates AKT-PI3K-mTOR signaling pathway through reducing the expression of PTEN, depending on autophagy in human liver cancer. The molecular mechanisms that LncRNAs regulate PKM2 expression via PTEN and PI3K/AKT/mTOR signal pathways in mediating tumor growth are briefly summarized in Figure 2.

**Figure 3:**
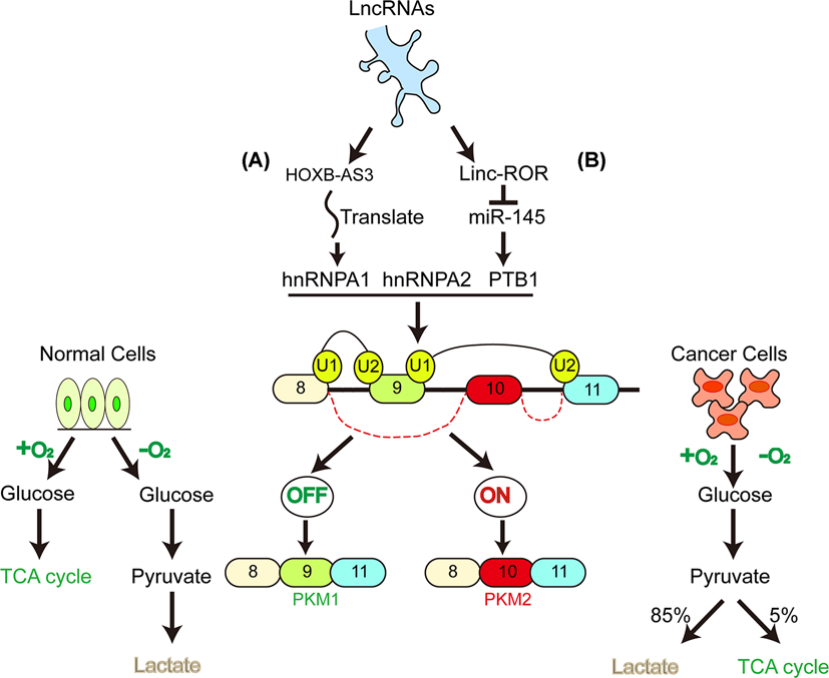
HnRNPA1, hnRNPA2 and PTB regulated differential splicing of PKM HnRNPs inhibit the cleavage of PKM mRNA by binding to exon 9, resulting in exon 9 exclusion and exon 10 inclusion and then generating PKM2. (**A**) The HOXB-AS3 peptide blocks hnRNPA1-mediated PKM splicing, thereby inhibits the formation of PKM2. (**B**) In contrast, lnc-RoR inhibits miR-145 and then reduces the switching of PKM1 to PKM2 via targeting PTBs. Switching from PKM1 to PKM2 promotes aerobic glycolysis and provides a selective advantage of normal cells to tumor formation.

Furthermore, the differential splicing of PKM, another unique feature different from regular pathways, plays a novel role in cancer cell metabolism. As stated above, hnRNPA1, hnRNPA2 and PTB regulate differential splicing of PKM. PTB regulates energy metabolism in cancer cells through selective splicing of PKM2. Both HIF-1α-mediated PKM gene transcription and selective cleavage of PKM precursor mediated by c-Myc and hnRNPs influence mTOR activation and tumorigenicity [62]. As the protooncogene and the transcription factor, c-Myc can up-regulate hnRNPs, promoting the expression of PKM2 in tumors. Moreover, HOXB-AS3 peptide attenuated the increase in PKM2 expression by overexpressing hnRNP A1. Thus, these precise molecular mechanisms used to define the roles of PKM splicing and subsequent PKM2 formation on tumor initiation and development provide a new direction for exploring the relationship between PKM2 and LncRNAs (Figure 3).

Finally, miRNAs, a class of endogenous non-coding single-stranded RNA molecules, bound by the 3′-UTR of a target messenger RNA with a protein-coding gene and mediate post-transcriptional gene expression [106]. Moreover, recent studies have proved that several LncRNAs regulate activities of miRNA through ceRNA mechanisms, which act as an endogenous ‘sponge’ [107]. In this review, we discussed several profound findings that LncRNAs, including Linc-ROR, MEG3 and HULC regulate activities of miRNA through ceRNA mechanisms and regulate the expression of PKM2.

## Perspectives

In summary, PKM2 has been confirmed that it is inextricably linked to the development and prognosis of human malignant tumors, particularly related to cancer cell metabolism. Investigating molecular mechanisms of LncRNAs targeting PKM2 is becoming a newly recognized hot research area. Exploring the role of PKM2-associated LncRNAs in cancer cell metabolism provides a great potential for developing novel strategy to block cancer development by targeting.

Interestingly, William et al. demonstrated that lack of PKM2 can promote breast cancer progression in a Brca1-loss-driven model [108]. However, accumulating evidence suggests that PKM2 is not required for the growth or progression of most tumors, based on the recent work that mice deficient of PKM2 exhibit enhanced tumorigenesis in several experimental models [109–112]. A recent study also found that PKM2 is not required for initiation or growth of pancreatic ductal adenocarcinoma tumors arising in the KP^−/−^C pancreatic cancer model [108]. These findings are consistent with previous results in that medulloblastoma tumors [111], colon tumor [113] and small cell lung cancer (SCLC) growth [114] do not require PKM2. Therefore, the application of PKM2 in targeted therapy of tumor has been questioned. However, due to its unique expression and regulatory mode, it remains an attractive focus in cancer research.

## Abbreviations

AKT: protein kinase B
ARF6: ADP-ribosylation factor 6
ceRNA: competitive endogenous RNA
CRC: colorectal cancer
ER: estrogen receptor
ERK: extracellular signal-regulated kinase
FEZF1-AS1: FEZF1 antisense RNA 1
HCC: hepatocellular carcinoma
HIF-1α: hypoxia inducible factor-1α
HOXB: Homeobox b gene
HOXB-AS3: HOXB cluster antisense RNA 3
HULC: highly up-regulated in liver cancer gene
IDH1: Isocitrate dehydrogenase 1
LincRNA: long intergenic non-coding RNA
linc-RoR: lncRNA regulator of reprogramming
LncRNA: long noncoding RNA
MEG3: maternally expressed gene 3
mTOR: mammalian target of rapamycin
O-GlcNAc: O-linked N-acetylglucosamine
PAK2: p21-activated kinase 2
PDAC: pancreatic ductal adenocarcinoma
PEP: phosphoenolpyruvate
PFK1: phosphofructokinase 1
PK: pyruvate kinase
PKC: protein kinase C
PKM2: M2 isoform of PK
PI3K: phosphatidylinositol 3-kinase
PTB: polypyrimidine tract binding
PTEN: phosphate and tension homology deleted on chromsome ten
ROS: reactive oxygen species
TGF-β: transforming growth factor β
TP53TG1: tumor protein 53 target gene 1
UTR: untranslated region
